# The Impact of Informal Caregiving on Patient-Reported Outcomes, Psychological Well-Being and Quality of Life in Inflammatory Bowel Disease: A Systematic Review

**DOI:** 10.3390/nursrep16030097

**Published:** 2026-03-13

**Authors:** Fabrizio Benedetti, Giulia Imperatori, Valeria Amatucci, Alessio Lo Cascio, Simone Amato, Daniele Napolitano

**Affiliations:** 1 Department of Specialist and Oncology Surgery, “S. Maria” Hospital, University of Perugia, 05100 Terni, Italy; fabribenedetti@libero.it; 2 Department of Sensory Organs and Andrology and Urogynecology Clinic, “S. Maria” Hospital, University of Perugia, 05100 Terni, Italy; 3 Department of Biomedicine and Prevention, University of Rome Tor Vergata, 00133 Rome, Italy; 4 Internal Medicine Research Unit, Policlinico Campus Biomedico, 00128 Rome, Italy; g.imperatori@policlinicocampus.it; 5 CEMAD-Fondazione Policlinico Gemelli IRCCS, 00168 Rome, Italy; valeria.amatucci@policlinicogemelli.it; 6 Direction of Health Professions, La Maddalena Cancer Center, 90146 Palermo, Italy; 7 Cardiac Intensive Care Unit, Heart Transplant Centre and ECMO Service, Azienda Ospedaliera San Camillo Forlanini, 00152 Rome, Italy; 8 SITRA and Scientific Direction-Fondazione Policlinico Gemelli IRCCS, 00168 Rome, Italy; daniele.napolitano@policlinicogemelli.it

**Keywords:** inflammatory bowel disease, caregivers, patient-reported outcomes, quality of life, health-related quality of life, psychological well-being, dyadic care

## Abstract

**Background/Objectives**: While caregiver burden in Inflammatory Bowel Disease (IBD) is well documented, the association between informal support and patient-reported outcomes (PROs), particularly health-related quality of life (QoL) and psychological well-being, remains underexplored. This systematic review synthesizes evidence on the association of informal caregiving on patient-reported QoL and psychosocial outcomes and maps the available evidence on clinical outcomes. **Methods**: Following international reporting guidelines and prospective protocol registration, a systematic search was conducted across five electronic databases between May and October 2025. Observational studies in adults with IBD assessing informal support and patient-reported or psychosocial outcomes were included. Owing to substantial heterogeneity in constructs and outcome measures, results were synthesised using a structured Synthesis Without Meta-analysis (SWiM) approach. Methodological quality was assessed using standardised critical appraisal checklists. **Results**: Six cross-sectional studies involving 1036 patients and 417 informal caregivers met the inclusion criteria. All studies reported a positive direction of association between higher levels or better quality of informal caregiver support and improved patient-reported QoL. Several studies identified psychological and relational factors, such as lower patient psychological distress and caregiver-related positive feelings and caring ability, as mechanisms statistically associated with this relationship. **Conclusions**: Available cross-sectional evidence suggests a positive association between informal support and patient-reported QoL/psychological outcomes in IBD, but causality cannot be inferred. Priorities include longitudinal dyadic studies and caregiver-inclusive interventions, alongside standardised definitions and measures of support.

## 1. Introduction

Inflammatory bowel diseases (IBD), including Crohn’s disease (CD) and ulcerative colitis (UC), are chronic relapsing inflammatory conditions of the gastrointestinal tract with a relapsing-remitting course [1,2]. Their pathogenesis involves a complex interplay of genetic, environmental, and behavioral factors such as diet and smoking that may contribute to inappropriate immune responses and long-term mucosal damage [3,4,5]. Over the past few decades, the incidence and prevalence of IBD have steadily increased, positioning these conditions as an increasing global health challenge [6,7,8]. Epidemiological projections suggest a continued rise in prevalence in the coming years [9,10]. IBD is a major and increasing health burden. GBD 2021-based estimates indicate that approximately 3.8 million people were living with IBD worldwide in 2021, with Western Europe remaining a high-burden region. In Europe, about 0.2% of the population is currently estimated to live with IBD [7,11,12]. In Italy, population-based estimates indicate a substantial and increasing burden, including approximately 442.3 prevalent cases per 100,000 inhabitants in a large Northern Italian area and projections of >15,000 new cases/year and ~260,000 prevalent cases nationally [13,14]. Consequently, IBD represents a substantial burden on healthcare systems. Beyond the economic impact, the disease significantly compromises patients’ quality of life (QoL), necessitating continuous monitoring and targeted therapeutic strategies.

The clinical presentation includes both gastrointestinal and extraintestinal manifestations, imposing a heavy physical and psychological toll [15]. Although flare occurrence cannot be predicted with complete accuracy, relapse risk may be influenced by multiple clinical, behavioural, and psychosocial factors. This uncertainty, together with the chronic nature of the disease, is often associated with anxiety, depression, and social isolation in patients and may also affect informal caregivers [16]. In this complex scenario, the role of the informal caregiver is crucial. Informal caregivers, typically family members or friends, provide long-term support for individuals with chronic conditions [17,18]. Their contribution extends beyond instrumental support, and may support patient self-care—the individual’s ability to independently manage their condition [19]. Recent studies indicate that positive emotions experienced by caregivers, such as satisfaction, gratitude, and family bonding, have been associated with more adaptive caregiver experiences. These emotions can alleviate caregiver burden and co-occur with better outcomes for both members of the dyad that enhances the well-being of both parties [20,21,22]. Specifically, within IBD, stronger positive feelings among caregivers have been associated with better health-related quality of life (HRQoL) in patients [21]. This perspective reinforces the conceptualization of the patient and caregiver as an interdependent dyad, aligning with the Theory of Dyadic Illness Management. This theoretical framework emphasizes that disease management is a shared phenomenon in which patients and caregivers adopt joint behaviors to manage health challenges, mutually influencing one another throughout the disease trajectory [19,23,24]. However, the majority of existing literature has prioritized the burden of caregiving, documenting its physical, emotional, social, and financial consequences [25,26,27,28]. Evidence regarding the active role of caregivers in improving clinical outcomes in IBD remains limited and heterogeneous. While some studies suggest that excessive caregiver burden can reduce QoL for both dyad members [28,29], others highlight the protective role of caregiver resilience and coping strategies, which sustain hope and adjustment despite high levels of burden [30]. Research in other chronic conditions has demonstrated that greater caregiver contribution to self-care improves treatment adherence and HRQoL, reduces depression, and even influences mortality outcomes [31,32,33,34,35]. Within the context of IBD, however, available evidence has predominantly focused on patient-reported outcomes (PROs) and psychosocial outcomes, while data on objective clinical endpoints remain limited. This imbalance represents a significant gap in current knowledge. Although clinical outcomes such as hospitalization rates, disease relapse, and treatment adherence are conceptually relevant in the context of informal caregiving, the existing literature in IBD predominantly focuses on PROs, particularly health-related QoL and psychological well-being. To date, evidence on the association of informal caregiving on objective clinical endpoints in IBD remains scarce, fragmented, and limited primarily to cross-sectional observations.

Alongside informal caregivers, the role of the IBD nurse is increasingly recognized as pivotal in contemporary models of care for IBD patients [36,37,38]. Beyond clinical support, the IBD nurse acts as a key facilitator of self-care, patient and caregiver empowerment, and health literacy, contributing to greater continuity of care and improved integration between formal and informal support systems [39]. By providing structured education and emotional support to both patients and caregivers, the IBD nurse may indirectly influence PROs, including QoL and psychological well-being, and mitigate caregiver burden through more effective dyadic interactions [40].

Therefore, the objective of this systematic review is to synthesise evidence on the association of informal caregiver support on PROs and psychosocial outcomes, such as QoL and psychological well-being, and to systematically map the available evidence on objective clinical outcomes among IBD patients when reported (e.g., hospitalisation, relapse, treatment adherence), thereby identifying evidence gaps. Given that the available literature is predominantly cross-sectional, this review aims to characterise reported associations, summarise potential explanatory variables described in the included studies, and identify gaps, particularly the absence of longitudinal and interventional evidence to inform priorities for future research and caregiver-inclusive models of care.

## 2. Materials and Methods

### 2.1. Methodology of the Review and Protocol Registration

This systematic review was conducted and reported in accordance with the PRISMA 2020 guidelines [41]. The protocol was registered in the International Prospective Register of Systematic Reviews (PROSPERO) with the registration number (CRD: 420251174936).

### 2.2. Research Question

In this review, an informal caregiver is defined as a family member, partner, or friend who provides unpaid assistance to an adult IBD patient. The research question was formulated using the PICO framework (Population, Intervention, Comparison, Outcomes) to specifically address the aim of the review based on a preregistered protocol [42]. Following an extensive literature search, no studies were found that compared patients with caregivers to patients without any informal support. Therefore, the framework was adapted to reflect the available evidence. The following elements were considered: Population (P): Studies including adult patients (≥18 years) with a confirmed diagnosis of IBD, including CD or UC. Intervention (I): Presence and quality of support provided by an informal caregiver. Comparison (C): IBD patients who receive reduced, insufficient, or qualitatively inadequate informal support, characterized by low perceived support [43] or limited involvement from the partner or family member. This also includes situations in which the caregiver is absent [44]. Caregiver burden was extracted as a contextual factor when reported. Outcomes (O): The primary outcome consists of patient-reported QoL and psychosocial health, assessed using validated instruments such as The Short Form 36 Health Survey [45,46] or disease-specific tools such as the Inflammatory Bowel Disease Questionnaire (IBDQ) [47]. Secondary outcomes included clinical and care-related indicators (e.g., treatment adherence, hospitalization or relapse rates, patient satisfaction); however, these were retained in the framework to comprehensively map the literature, acknowledging that their empirical assessment depended on data availability in the included studies.

### 2.3. Search Strategy

A comprehensive and systematic literature search was conducted between May 2025 and October 2025 in the following scientific databases: PubMed, CINAHL, Web of Science, Scopus, and the Cochrane Library. The full search strategy is available in Supplementary Data S1. To obtain a targeted and precise search, a combination of keywords and MeSH terms was used, appropriately combined with Boolean operators. Initially, all retrieved citations were imported into the Rayyan software (https://rayyan.com/), where duplicates were removed. Two reviewers independently and blindly conducted the screening of titles, abstracts, and full texts. Any conflicts at each stage of the process were resolved through discussion or, if necessary, by a third reviewer serving as arbitrator and making the final decision.

### 2.4. Inclusion Criteria

Quantitative observational studies (cross-sectional, cohort, case–control) that explored the impact of informal caregiver support on PROs, including QoL and psychosocial health, in adult IBD patients were included. Studies examining clinical outcomes were also considered eligible, provided they were present in the existing literature. Although the review primarily focused on PROs, caregiver-related psychosocial outcomes (e.g., caregiver burden) were also considered eligible when they were directly linked to informal caregiving processes relevant to the patient’s care context and disease management. Studies on pediatric populations, secondary literature (reviews, meta-analyses), studies in which the impact of the caregiver could not be isolated, and records for which the full text was not accessible were excluded. One included study enrolled mixed-age patient–caregiver dyads; however, it was retained as it focused exclusively on caregiver burden as a contextual factor and did not contribute to PROs estimates.

### 2.5. Risk of Bias and Methodological Quality Assessment of the Studies

The risk of bias and methodological quality of the included articles were independently assessed by two reviewers, with any conflicts resolved through discussion or by a third reviewer. Because the included studies were cross-sectional, the JBI Critical Appraisal Checklist for Analytical Cross-Sectional Studies was used (JBI Critical Appraisal Tools accessed from https://jbi.global/critical-appraisal-tools [accessed on 1 October 2025]). This tool evaluates eight domains to determine the validity of a study, with particular attention to: (1) the clarity of the sample inclusion criteria; (2) the validity and reliability of the measurements of exposure (support) and outcome (QoL); (3) the identification and management of key confounding factors. We report item-level judgments and the proportion of criteria met per study; we avoid interpreting total scores as definitive quality labels. The quality of each study was classified as high (>70%), moderate (50–69%), or low (<50%) based on the percentage of criteria met. This structured process ensured that the synthesis was based on the most methodologically robust studies [48]. The results of this assessment are reported in Supplementary Data S2.

### 2.6. Assessment of Evidence Certainty

The overall certainty of the evidence was mapped using the Oxford Centre for Evidence-Based Medicine (OCEBM) 2011 framework [49], the details of which are reported in Supplementary Data S3. Given the inclusion of only cross-sectional studies, the evidence synthesized in this review falls under Level of Evidence 4. According to the OCEBM hierarchy, this level is appropriate for studies that, by measuring exposure and outcome simultaneously, can identify strong associations but cannot establish causality due to the lack of a temporal sequence. This classification further supports the interpretation of findings as associative and primarily applicable to PROs.

### 2.7. Data Extraction

The data from the selected articles were extracted and reported in the table (Table 1), which includes: Author, Year, Country, Type of study, Sample size, Objectives, Role of Caregiver, Effect of caregiving, Key Finding. Outcomes were categorized a priori as patient-reported (e.g., quality of life, psychological symptoms) or clinical, to facilitate a transparent synthesis of the available evidence.

### 2.8. Data Synthesis

Due to significant clinical and methodological heterogeneity, particularly in the variety of instruments used to measure both caregiver support and patient QoL, a statistical meta-analysis of the data was not considered appropriate. A structured narrative synthesis was therefore conducted, following the recommendations of the SWiM (Synthesis Without Meta-analysis) guidelines [50]. To ensure a transparent and replicable process, the synthesis was carried out through a multi-stage thematic analysis. The first phase involved data extraction and familiarization: the key findings of each study were read repeatedly to gain a thorough understanding of the evidence base. In the second phase, involving coding and grouping, the results were aggregated based on conceptual similarity. For example, findings related to “reduction of anxiety” [51] and decreased catastrophizing [43] were grouped under the code “psychological impact on the patient.” Finally, through an iterative process, the codes were organized and refined into overarching themes, which represent the main patterns identified in the data. This process led to the definition of three themes structuring the results: the positive association between support and QoL, mediating mechanisms, and contextual moderating factors. Managing the heterogeneity of instruments represented a guiding principle for the synthesis: since numerical values could not be aggregated, the analysis focused on the direction, coherence, and significance of the associations reported across studies. Where clinical outcomes were theoretically relevant but empirically absent, this was explicitly documented as a finding of the review. This thematic synthesis is presented narratively in the results section and summarized in Table 2, providing a solid evidence base for future research and the development of targeted psychosocial interventions.

**Table 1:** Data extraction.

Author, Year	Country	Type of Study	Characteristics of Sample Size/Population	Objectives	Role of Caregiver	Effect of Caregiving	Key Finding
Fang et al., 2022 [21]	China	Cross-sectional study	Patients (n = 181): mean age 34.9 ± 11.4; male 56%, female 44%; CD 53.6%, UC 46.4% Caregivers (n = 181): mean age 43.7 ± 13.2; male 36%, female 64%; age groups 18–44: 50%, 45–60: 35%, >60: 13% (2% missing)	To explore the association between caregiver “caring ability,” positive feelings, dyadic closeness, and patient QoL	Informal/primary caregiver.	Caregiver positive feelings are correlated with better patient HRQoL. Caregiver “caring ability” mediates this association (explaining 34.1% of the variance). Relational closeness is also positively correlated with patient HRQoL.	Caregiver positive feelings and caring ability mediate the relationship between dyadic closeness and the patient’s quality of life.
Fu et al., 2020 [51]	China	Cross-sectional study	Patients (n = 199): mean age 35.3 ± 10.6; male 57.3%, female 42.7%; CD 56.8%, UC 43.2%	To examine the mediating effect of psychological symptoms on the relationship between disease activity, social support, and HRQoL.	Source of social support (family, friends, others).	Social support is positively correlated with HRQoL (r = 0.338, *p* < 0.01). Patient psychological symptoms (anxiety and depression) fully mediate this relationship (indirect effect: β = 1.20, *p* < 0.001).	The association between social support and HRQoL is explained by the capacity of support to alleviate the patient’s psychological distress.
Katz et al., 2016 [43]	Canada	Cross-sectional study	Patients (n = 164): mean age 47.28 ± 16.1 (range 18–81); male 44.5%, female 55.5%; CD 57.9%, UC 37.8%, indeterminate/both 4.3%; partnered: 66.5%	To examine the relationship between social support and QoL in IBD patients and identify mechanisms, including cognitive factors (catastrophizing and optimism) as mediators.	Source of social support (significant other, family, friends).	Spouse responses (solicitous, negative, distracting) were measured. Lower perceived support and higher negative responses from the spouse predicted worse QoL. Patient Helplessness (a subscale of catastrophizing) fully mediated the relationship between negative spouse responses and QoL.	Social interaction variables are associated with IBD-related QoL, but the patient’s experience of Helplessness reduces their ability to benefit from social support. Interventions improving social interactions and reducing catastrophizing are beneficial.
Lahat et al., 2014 [52]	Israel	Cross-sectional study	Patients (n = 101): mean age 45.36 ± 15.46; men 53, women 48; CD 62, UC 39	To assess patients’ opinions regarding sharing information with their partners and their partner’s involvement in disease management.	Partner/Spouse.	The vast majority of patients desired greater partner involvement. 93% shared health problems, and 88% believed that greater partner involvement would help them better manage their disease.	IBD Patients perceive their partner’s involvement as a crucial factor for better disease management and desire a more active role from them.
Maunder et al., 2007 [44]	Canada	Cross-sectional study	Patients (n = 155, UC only): (women 52.9% in single/separated vs 39.4% in married/common-law); marital status single/separated/divorced 32.9%, married/common-law 67.1%; disease status at assessment active UC n = 20, remission n = 135.	To determine whether the perceived impact of UC on daily activities (illness intrusiveness) is greater for unmarried individuals compared to those in a relationship.	Implicit: Spouse/Partner defined via marital status.	Patients who were single or separated reported significantly greater illness intrusiveness than married/cohabiting patients (*p* = 0.02), even after controlling for age, income, and social support.	Single marital status and younger age are independent risk factors for a greater perceived functional impact of the disease.
Yuan et al., 2025 [53]	China	Cross-sectional study	Patients (n = 236): mean 32.11 ± 11.39; male 63.98%, female 36.02%; UC 24.15%, CD 75.85%	To assess the current state of caregiver burden in IBD and identify associated factors.	Primary caregiver (family members).	Evidence on caregiver-related psychosocial burden within the informal caregiving context and was interpreted as part of the dyadic caregiving framework, rather than as a study of objective patient clinical outcomes. Caregivers of IBD patients experience a substantial and multidimensional burden. Higher caregiver burden is associated with elevated levels of anxiety, depression, and poor sleep quality.	Predictors of increased caregiver burden include female caregiver gender, younger patient age, and greater disease severity. Caregiver burden is a significant phenomenon that negatively impacts caregiver well-being.

Note: CD, Crohn’s Disease; IBD, Inflammatory Bowel Disease; HRQoL, Health-Related Quality of Life; QoL, Quality of Life; UC, Ulcerative Colitis; Dyad, the patient–caregiver pair considered as a single unit.

**Table 2:** Thematic synthesis of results regarding informal caregiver support and outcomes in IBD patients.

Theme	Supporting Studies	Key Findings
Positive association between caregiver support and patient Quality of Life	[21,43,44,51]	The presence of general social support, a stable partner, and high perceived spousal support are significant predictors of better QoL and HRQoL in IBD patients.
Mediating role of the patient’s psychological status	[43,51]	Caregiver support improves QoL by acting as a protective factor against the psychological burden of the disease. The association is mediated by the reduction of the patient’s psychological symptoms (anxiety, depression) and by the decrease in Helplessness catastrophizing.
Caregiver-related mediating mechanisms	[21]	The relationship between dyadic closeness and the patient’s HRQoL is mediated by the caregiver’s positive feelings and “caring ability”. This pathway explains a significant portion (34.1%) of the variance in QoL.
Impact of caregiving burden on support effectiveness	[44,53]	A high caregiver burden (associated with anxiety, depression, and poor sleep quality) represents an obstacle to the ability to provide effective support. The burden is greater when the patient is young and the disease is more severe.
Quality of interaction as a key modulator	[43]	The quality of the interaction is crucial. Negative or irritable responses from the spouse are directly associated with worse patient QoL, negating the potential benefits of support.
Patient perception and need for support	[52]	Patients perceive partner involvement as a crucial factor for better disease management. The overwhelming majority (88%) desire a more active role and greater involvement from their partner.

Note: The table summarizes thematic areas and evidence derived from included studies. It provides a narrative synthesis in line with SWiM recommendations for systematic reviews without meta-analysis. IBD, Inflammatory Bowel Disease; HRQoL, Health-Related Quality of Life; QoL, Quality of Life.

## 3. Results

### 3.1. Study Selection and General Characteristics

**Figure 1:**
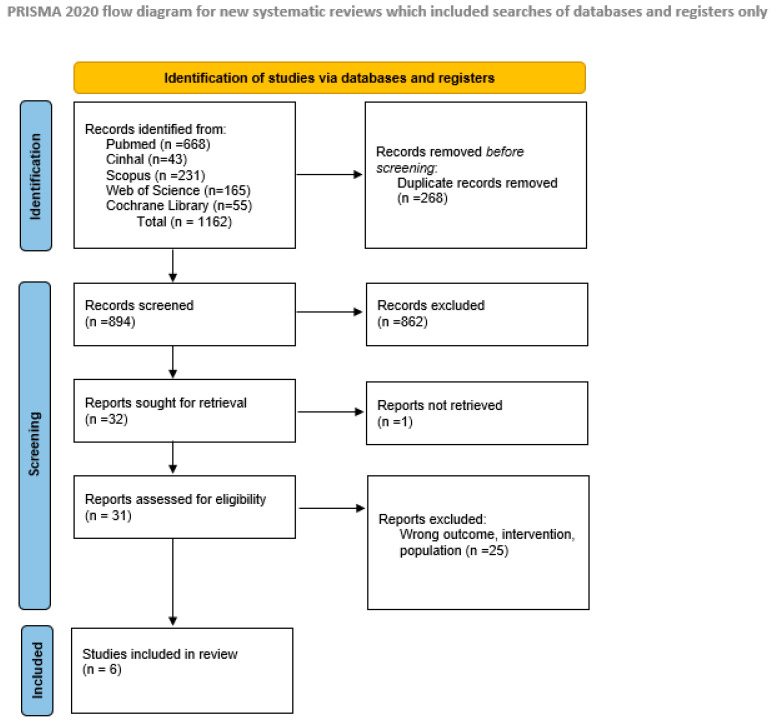
PRISMA flowchart of the study selection process. The diagram illustrates the systematic search strategy, the number of records identified across five electronic databases, the screening process, and the final number of cross-sectional studies included in the review.

Electronic database searches identified a total of 1162 records (PubMed: 668; CINAHL: 43; Scopus: 231; Web of Science: 165; Cochrane Library: 55). After removal of 268 duplicates, 894 records were screened by title and abstract, of which 862 were excluded. Thirty-two full-text articles were assessed for eligibility. One report could not be retrieved, and 25 studies were excluded for failing to meet the inclusion criteria (e.g., wrong population, intervention, or outcome). Ultimately, six studies were included in the qualitative synthesis. The study selection process is summarised in the PRISMA flow diagram (Figure 1).

All included studies employed a cross-sectional design (n = 6). The cumulative sample comprised 1036 IBD patients and 417 informal caregivers. Detailed study characteristics are reported in Table 1.

Methodological quality was assessed using the JBI Critical Appraisal Checklist for Analytical Cross-Sectional Studies (Supplementary Data S2). Two studies (33.3%) achieved the maximum quality score [21,53], while 3 studies (50%) obtained a score of 75% [43,44,51] and one study (16.7%) scored 62.5% [52].

Overall methodological quality was moderate to high (mean score: 81.3%; range: 62.5–100%). The most frequently identified limitation concerned insufficient reporting or management of potential confounding factors.

All included studies reported patient-reported and psychosocial outcomes; no eligible study examined objective clinical endpoints such as hospitalisation, disease relapse, or treatment adherence.

### 3.2. Thematic Summary of Results

Based on the narrative synthesis of the included studies and in line with the SWiM guidelines, several key themes emerged regarding the relationship between caregiver support and outcomes in IBD patients. These themes are summarized in Table 2 and detailed below. As all included studies were cross-sectional, the results are inherently limited to the analysis of associations at a single point in time, precluding the establishment of causal links. To address the primary objective, results were organized thematically to explore: the association between caregiver support and patient QoL; the psychological and behavioral mechanisms mediating this relationship; and caregiver characteristics, including burden. The evidence demonstrates that informal caregiver support is not only associated with patient-reported QoL among IBD patients but also mediated by specific psychological mechanisms and modulated by key contextual factors.

### 3.3. Structure of the Synthesis and Standardised Metric

Given the heterogeneity of caregiver-related constructs, outcome measures, and analytical approaches, statistical meta-analysis of effect estimates was not appropriate. Results were synthesised using alternative methods in accordance with the SWiM reporting guideline. Studies were grouped for synthesis according to conceptual similarity into three predefined synthesis groups: associations between informal caregiver support and patient-reported QoL; psychological and behavioural mechanisms mediating these associations; contextual and relational factors modulating the effectiveness of caregiver support. Although clinical outcomes were included in the eligibility criteria, no study reported objective disease-related endpoints. This group was retained to explicitly document the absence of evidence.

Across all synthesis groups, the standardised metric was the direction of effect, defined relative to outcomes, with higher values indicating better patient status (e.g., higher QoL or lower psychological distress). Direction of effect was classified as positive, negative, null, or unclear. Statistical significance and effect estimates were reported when available but were not pooled. All six included studies reported a positive direction of association between informal caregiver support and patient-reported QoL [21,43,44,51,52,53]. Caregiver support was operationalised using heterogeneous constructs, including perceived social support [51], spousal or partner support [43], relational closeness [21], and marital or partnership status [44]. Higher levels of perceived social support were associated with better QoL among IBD patients [51]. Similarly, greater perceived spousal support [43] and the presence of a stable partner [44] were associated with higher disease-specific or generic QoL scores. Fang et al. further specified that caregiver positive feelings and perceived relational closeness were positively associated with patient health-related QoL [21]. Despite variability in measurement instruments and analytical strategies, the direction of association between caregiver support and QoL was consistent across all studies. In multivariable analyses, caregiver-related variables remained associated with QoL after adjustment for sociodemographic and disease-related covariates [43,51,53].

### 3.4. Mediating Mechanisms Linking Caregiver Support to Quality of Life

Quantitative details of the mediation models and effect estimates underlying the associations between informal caregiver support and patient-reported QoL are summarized in Table 3. Four studies explored mediating mechanisms underlying the association between caregiver support and patient-reported QoL [21,43,51,53]. Two complementary pathways emerged. The first pathway involved patient psychological factors. Fu et al. demonstrated that anxiety and depression fully mediated the relationship between perceived social support and QoL, indicating that the association operated primarily through reduced psychological distress [51]. Katz et al. identified helplessness catastrophizing as the only significant cognitive mediator linking spousal variables to patient QoL, suggesting that specific illness-related appraisals may undermine patients’ ability to benefit from available support [43]—the second pathway involved caregiver-related behavioural mechanisms. Fang et al. tested a sequential mediation model in which relational closeness was associated with positive caregiver feelings, which in turn were associated with greater caring ability; increased caring ability acted as the final mediator leading to higher patient health-related QoL. This mediation pathway accounted for 34.1% of the total variance in QoL scores [21]. All mediation analyses were cross-sectional and are therefore interpreted as associative rather than causal mechanisms.

### 3.5. Contextual and Relational Factors Modulating the Effectiveness of Support

The effectiveness of caregiver support varied according to relational quality and caregiver well-being. Katz et al. reported that adverse spousal responses, such as irritation or criticism, were independently associated with poorer patient QoL, whereas solicitous or distracting responses were not [43]. These findings suggest that dysfunctional dyadic interactions may negate or reverse the potential benefits of support. Caregiver burden emerged as a key contextual factor limiting caregiving effectiveness. Higher burden levels were associated with increased caregiver psychological distress, including anxiety, depression, and sleep disturbances [53]. Caregiver burden was greater in contexts of higher disease severity and younger patient age [53]. In parallel, younger patients reported greater illness intrusiveness [44], a condition associated with increased caregiver burden, suggesting a pattern of reciprocal vulnerability within the caregiving dyad. Patients’ perspectives corroborated the relevance of informal caregiving, with 88% reporting that greater partner involvement would facilitate disease management [52].

### 3.6. Absence of Evidence on Clinical Outcomes

No included study examined objective clinical outcomes such as hospitalisation, disease relapse, or treatment adherence. This absence was consistent across all databases and screening stages and reflects a systematic gap in the literature rather than selective reporting.

### 3.7. Certainty of the Evidence

According to the Oxford Centre for Evidence-Based Medicine framework, the overall certainty of evidence was classified as Level 4. Although the direction of associations was consistent across studies for patient-reported QoL and psychosocial outcomes [21,43,44,51,52,53], the exclusive reliance on cross-sectional designs limits causal inference and temporal interpretation.

**Table 3:** Quantitative associations between informal caregiver support and PROs in IBD (SWiM synthesis).

Study	Sample (Patients/Caregivers)	Caregiver-Related Exposure	Outcome (Instrument)	Analysis	Effect Estimate	95% CI	*p*-Value	Direction of Association	Notes
Fang et al., 2022 [21]	181/181	Caregiver positive feelings; caring ability	HRQoL (IBDQ)	Sequential mediation (SEM)	Indirect effect β = 0.24	0.09 to 0.44	<0.001	Positive	Relational closeness → positive feelings → caring ability → HRQoL; 34.1% variance explained
Fu et al., 2020 [51]	199/–	Perceived social support	HRQoL (IBDQ)	Multivariable regression + mediation	β = 1.38	0.82 to 1.93	<0.01	Positive	Effect fully mediated by anxiety/depression (HAD). Social support improves QoL by reducing psychological distress.
Katz et al., 2016 [43]	164/–	Perceived spousal support	QoL (S-IBDQ)	Multivariable regression	β = 0.31	Not reported	<0.01	Positive	Helplessness catastrophizing identified as key mediator between support and QoL
Katz et al., 2016 [43]	164/–	Negative spousal responses	QoL (S-IBDQ)	Multivariable regression	β = −0.41	Not reported	<0.01	Negative	Adverse relational behaviours independently associated with poorer QoL
Maunder et al., 2007 [44]	155/–	Marital/partner status	Illness intrusiveness	ANCOVA	F = 5.73	Not reported	0.02	Positive	Unmarried patients in remission reported illness intrusiveness similar to those with active disease. Age and social support also significant
Yuan et al., 2025 [53]	236/236	Caregiver Anxiety (SAS) & Depression (SDS)	Caregiver Burden (CBI total score)	Multiple linear regression	R^2^ = 0.645; β = 0.329–0.453	0.136 to 0.709 (for β)	<0.01	Positive	Model explains 64.5% of variance in burden. Female caregivers and those with longer caregiving hours reported significantly higher burden.
Lahat et al., 2014 [52]	101/–	Partner involvement	Patient-reported coping	Descriptive + χ^2^	OR not reported	Not reported	<0.001	Positive	88% of patients believed partner involvement helped them cope better with disease; 70% wanted partners more involved

Note: IBD = Inflammatory Bowel Disease. SWiM = Synthesis Without Meta-analysis, a methodological approach for the structured narrative synthesis of quantitative findings when meta-analysis is not feasible. HRQoL = Health-Related Quality of Life. QoL = Quality of Life. IBDQ = Inflammatory Bowel Disease Questionnaire. S-IBDQ = Short Inflammatory Bowel Disease Questionnaire. SEM = Structural Equation Modeling. β = non-standardized regression coefficient (B). CI = Confidence Interval (95%). HAD = Hospital Anxiety and Depression Scale. ANCOVA = Analysis of Covariance. F = F-statistic of the model. SAS = Self-Rating Anxiety Scale. SDS = Self-Rating Depression Scale. CBI = Caregiver Burden Inventory. R^2^ = coefficient of determination. χ^2^ = chi-square test. OR = Odds Ratio. *p*-values < 0.05 indicate statistical significance.

## 4. Discussion

This systematic review provides a synthesis of the available quantitative literature investigating the relationship between informal caregiver support and patient-reported health outcomes, particularly QoL and psychosocial well-being, in IBD patients. Evidence from the broader chronic illness literature suggests that the well-being, competence, and emotional burden of the caregiver significantly influence not only the patient’s QoL but also crucial clinical outcomes, such as therapeutic adherence and the overall course of the disease [54]. However, within the IBD literature synthesized in this review, evidence on objective clinical endpoints remains largely unavailable. In the specific context of IBD, constructs such as caregiver resilience and self-efficacy are emerging as key determinants of their active engagement in disease management [55].

**Figure 2:**
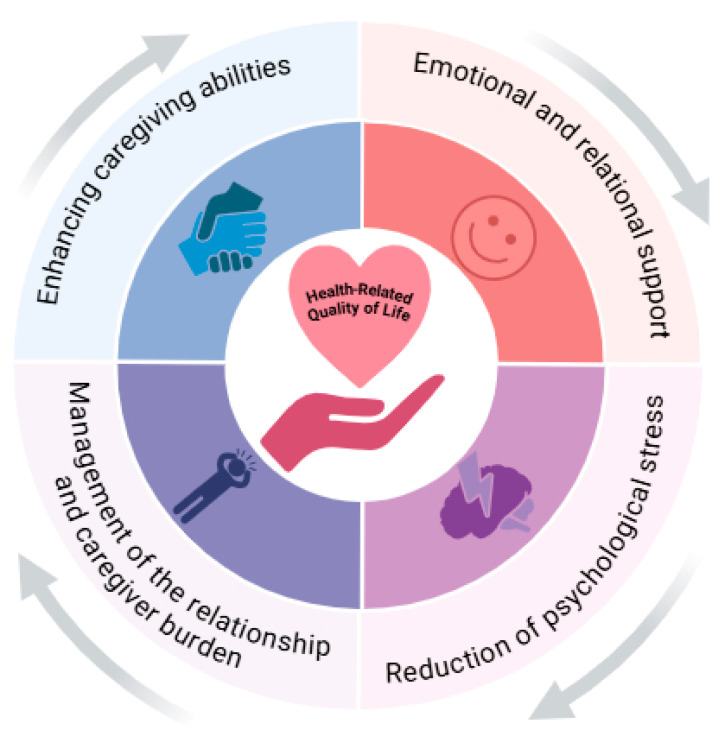
Conceptual framework of the impact of informal caregiving on inflammatory bowel disease outcomes. The figure synthesizes the dyadic pathways identified in the literature. Informal caregiver support improves patient quality of life through the mediation of reduced psychological distress and enhanced caregiver caring ability. This process is moderated by the level of caregiver burden.

To visualize these complex associations, Figure 2 synthesizes evidence from the six included studies, mapping direct associations, mediating psychological mechanisms (such as reduced catastrophizing and increased caring ability), and critically moderating the role of caregiver burden.

Importantly, this conceptual framework reflects patient-reported and psychosocial pathways identified in cross-sectional studies and does not imply validated causal relationships or effects on clinical outcomes. IBD affects not only the individual but also operates fundamentally as a shared stressor that deeply impacts the entire family unit. While the beneficial role of family support is intuitive, this review highlights a critical gap in the empirical evidence. The contribution of this study extends beyond merely confirming an association; it elucidates the underlying mechanisms driving these outcomes within the domain of patient-reported QoL. The fluctuating and unpredictable nature of the pathology [1,11,56] delineates a complex landscape that distinguishes caregiving in IBD from that in other chronic conditions. This distinction is crucial for interpreting the high levels of psychological distress and burden identified in our included studies [53]. Unlike neurodegenerative diseases, where the caregiving trajectory is often linear and progressive toward complete dependence [20], the IBD caregiver must navigate a cyclical landscape of unpredictable acute flares and remission. This specific unpredictability likely drives the anxiety and “helplessness catastrophizing” observed in our synthesis [43], requiring a constant state of vigilance that differs from other chronic conditions. However, our findings regarding the protective role of “caring ability” [21] suggest that this challenge can also foster resilience. This mirrors evidence from heart failure research, where perception of meaning and satisfaction in the caregiver role, rather than just the burden, is directly associated with better patient self-management outcomes [31,34].

In light of this review’s findings, the IBD nurse emerges as a strategic figure in translating evidence on informal support into structured and sustainable clinical practice [53]. Although the included studies primarily focus on the patient–caregiver relationship, the IBD nurse can enhance the effectiveness of this dyad by facilitating coping processes, communication, and shared responsibility in care [57]. Through early identification of caregiver distress and targeted educational and psychosocial interventions, the IBD nurse may strengthen dyadic functioning and indirectly improve PROs. Integrating this role systematically into IBD care pathways could therefore help bridge the gap between observed associations and evidence-based interventions, transforming informal support into a formal component of comprehensive, nurse-led models of care [58,59,60].

Although the protocol sought to evaluate the relationship between informal caregiving and QoL in IBD with attention to longitudinal implications, all eligible quantitative studies (n = 6) used cross-sectional designs. As a result, the absence of longitudinal evidence should be interpreted as a key finding of this review, reflecting a major gap in the existing literature. This finding is not a limitation, but the main result of the evaluation: the identification of a fundamental gap in IBD research. Current evidence establishes a strong associative link but, due to the lack of temporal sequence, cannot infer causation [48,49]. Moreover, this limitation confines the interpretation of findings to PROs, precluding conclusions regarding objective clinical endpoints.

Although our review found a consistent link between support and QoL, the specific caregiver behaviors that drive this association remain unclear. Research on other chronic conditions offers a more detailed perspective; studies on COPD, for example, have categorized caregiver contributions into the distinct domains of self-care maintenance, self-care monitoring, and self-care management [61,62], suggesting that future IBD research should adopt similar multidimensional frameworks. The findings of this synthesis align with the Theory of Dyadic Illness Management, supporting a paradigm shift in which the caregiver is viewed not merely as a support figure but as a co-actor whose competence constitutes a tangible therapeutic asset. Longitudinal research on other chronic conditions has elucidated this mechanism, demonstrating that caregiver contribution improves patient outcomes both directly and indirectly, by mediating patient self-care [63]. Essentially, an effective caregiver enhances the patient’s capacity for self-management, thereby optimizing health outcomes. Whether similar mechanisms translate into measurable clinical benefits in IBD remains to be empirically established. Concepts such as self-efficacy [64] and caregiver preparation [65], already identified as critical in other clinical populations, suggest that effective support relies less on the mere presence of a caregiver and more on promoting their internal psychological resources. Recent evidence specific to IBD confirms that caregiver resilience is positively associated with self-efficacy and active engagement in disease management [55], shifting the clinical focus from burden to empowerment [66]. However, providing such support entails a high personal cost. The included studies identified a substantial burden [53], outlining a potential “vicious cycle”: increased disease severity heightens caregiver burden, which may subsequently impair the caregiver’s “caring ability” [21], potentially leading to deteriorating patient QoL and indirectly affecting disease management. This dynamic appears particularly pronounced in vulnerable dyads, such as those including younger patients or those with CD [44,53].

### 4.1. Strengths and Limitations

The primary strength of this review lies in the high methodological quality of the process, including adherence to PRISMA 2020 guidelines, a registered PROSPERO protocol, and rigorous JBI critical appraisal. The narrative synthesis, structured according to the SWiM framework, allowed for the transparent integration of heterogeneous studies. However, the most significant limitation is the exclusively cross-sectional nature of the included studies. Consequently, applying the OCEBM framework, the entire body of synthesized evidence is classified as Level of Evidence 4. According to the OCEBM hierarchy, this level is appropriate for studies that, by measuring exposure and outcome simultaneously, can identify strong associations but cannot establish causation due to the lack of temporal sequence. Thus, while the results provide solid evidence of an association between caregiver support and patient-reported QoL and psychosocial outcomes, conclusions regarding causality and clinical endpoints must be interpreted with caution. This methodological limitation underscores the urgent need for future longitudinal research (Level 2) to confirm the directionality of this relationship [48]. Another limitation and potential source of bias we acknowledge is that we were unable to obtain the full text of one otherwise relevant manuscript, which may have reduced the completeness of the synthesis and introduced selection bias into included evidence. Furthermore, the certainty of the evidence is constrained by methodological shortcomings in some primary studies, such as the suboptimal management of confounding factors [43] and heterogeneity in the measurement of support [44].

### 4.2. Implications for Clinical Practice and Future Research

The findings of this review have potential and clinically relevant implications, as detailed in Table 4. From a clinical perspective, IBD management must evolve from a patient-centered model to one that treats the patient–caregiver dyad as a single unit of care. Given that current evidence predominantly concerns PROs, clinical interventions should primarily aim to improve QoL, psychological well-being, and dyadic functioning. Our synthesis indicates that the mere presence of a caregiver is insufficient; rather, the perceived effectiveness of support is associated with the quality of the dyadic interaction and the psychological resources of both parties. In this context, existing caregiver-focused frameworks developed in other chronic conditions, such as the CARE (Caregiver-Centered, Active Engagement, Reinforcement, Education) model, may offer a useful conceptual reference for the development of future IBD-specific interventions, although their effectiveness in this population remains to be empirically tested [54].

Regarding future research, there is an urgent need to conduct longitudinal and dyadic studies to disentangle causality and to determine whether improvements in PROs translate into measurable clinical benefits, as proposed by other protocols [19,60]. Furthermore, it is crucial to establish a conceptual and methodological distinction between the caregiver’s contribution to patient self-care and the caregiver’s own self-care. While this review focused on the former, the caregiver’s ability to maintain their own psychological and physical well-being actively remains a poorly explored area in IBD. The development of theoretically grounded instruments, such as the Self-Care of Informal Caregivers Inventory (SC-ICI), now offers the opportunity to measure this construct systematically [67]. Validating and applying such instruments in the context of IBD represents a research priority to fully understand caregivers’ needs and develop targeted interventions to preserve their health and the sustainability of their caregiving role. Finally, future studies should systematically map the specific needs of IBD caregivers [68] and analyze diverse typologies of dyadic interactions to personalize interventions [69].

**Table 4:** Implications for clinical practice.

Category	Implications for Clinicians	Implications for Patients and Caregiver
Dyadic Assessment and Intervention	Integrate the assessment of patient–caregiver dynamics into routine clinical practice. Utilize screening questions to evaluate the quality of perceived support and communication. Offer joint psychoeducational interventions aimed at improving communication and dyadic coping strategies.	Engage in open, honest communication about needs, fears, and challenges related to the disease. Schedule regular “check-ins” to discuss how the illness is influencing the relationship and the well-being of both parties.
Screening and support for caregiver burden	Conduct proactive screening for burden, anxiety, and depression in informal caregivers, especially in severe illness settings or young patients. Provide caregivers with information about dedicated support resources, including support groups, psychological counseling, and respite care.	Recognize the signs of burnout (e.g., emotional exhaustion, irritability) and actively seek support. Express appreciation and acknowledge the caregiver’s effort to help mitigate their sense of burden.
Improving interaction quality	Educate dyads on the impact of negative interactions (e.g., criticism, irritation) on patient QoL and provide strategies for constructive communication. Encourage a focus on emotional and practical support, rather than distracting or minimizing responses.	Learn and practice active listening and communication techniques to navigate stressful periods. Collaborate to solve problems, framing the illness as a shared challenge rather than an individual burden.
Patient empowerment and caregiver engagement	Recognize and validate the patient’s desire for greater partner involvement, facilitating conversations on how the caregiver can assume a more active role in disease management. Encourage caregiver participation in medical appointments (with consent) to enhance shared understanding of the treatment plan.	Clearly express specific areas where help or involvement is desired. Actively seek information about the disease (Caregivers) to provide competent support and foster a sense of inclusion in the care pathway.

QoL, Quality of Life. Note: The evidence summarised in this table is derived from cross-sectional observational studies. Therefore, the reported associations should not be interpreted as causal and should be considered hypothesis-generating.

## 5. Conclusions

This systematic review establishes a robust, cross-sectional association between high-quality informal caregiver support and improved patient-reported QoL and psychosocial outcomes in IBD patients. The evidence suggests that specific psychological and behavioral mechanisms mediate this link, yet caregiving entails a high burden that may compromise sustainability. The absence of longitudinal data and objective clinical endpoints precludes the determination of directional causality and clinical effectiveness, identifying this as the primary gap to be addressed in future research. Ultimately, clinical management and research in IBD must shift toward a dyadic model that actively empowers caregivers, ensuring that this promising association translates into measurable, sustainable, and clinically meaningful outcomes.

## Data Availability

The data underlying this article are available in the article and in its online Supplementary Material.

## Supplementary Materials

The following supporting information can be downloaded at: https://www.mdpi.com/article/10.3390/nursrep16030097/s1, Supplementary Data S1. Search Strategies. Supplementary Data S2. Methodological Quality Assessment (JBI Critical Appraisal Checklist). Supplementary Data S3. Levels of Evidence (OCEBM).

## Public Involvement Statement

No public involvement in any aspect of this research.

## Guidelines and Standards Statement

This systematic review was conducted and reported in accordance with the PRISMA 2020 guidelines. The protocol was registered in the International Prospective Register of Systematic Reviews (PROSPERO) with the registration number (CRD: 420251174936).

## Abbreviations

The following abbreviations are used in this manuscript:

CD	Crohn’s Disease;
HRQoL,	Health-Related Quality of Life;
IBD	Inflammatory Bowel Disease;
JBI	Joanna Briggs Institute;
PRISMA	Preferred Reporting Items for Systematic Reviews and Meta-Analyses
PROs	Patient-Reported Outcomes
QoL	Quality of Life
SWiM	Synthesis Without Meta-analysis
UC	Ulcerative Colitis
